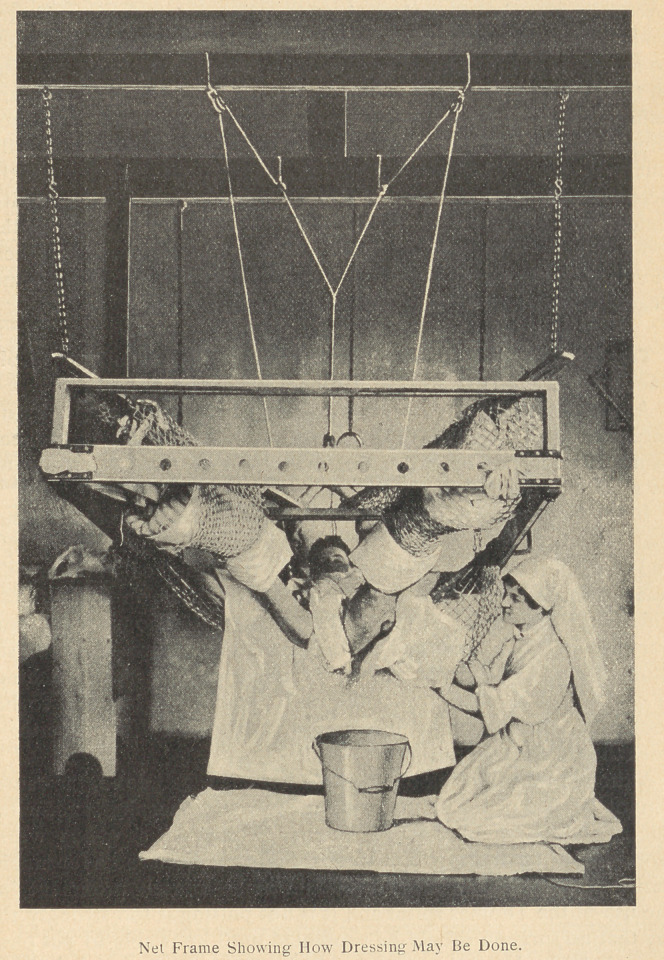# Treatment of Fractures of the Femur

**Published:** 1918-09

**Authors:** 


### WAR MEDICINE

Published by the American Red Cross Society in France
for the
Medical Officers of the American Expeditionary Forces

### RESEARCH SOCIETY REPORTS

### THE EIGHTH SESSION OF THE RESEARCH SOCIETY OF THE AMERICAN RED CROSS IN FRANCE

July 26 and 27, 1918, at the Hotel Continental, Paris.

*Major Walter B. Cannon, Chairman of the Research Committee, presided throughout the session.*

*The subject of the first meeting, Friday July 26, at 2 : 00 P. M., was " Treatment of Fractures of the Femur ". It was discusssed by Major Sinclair, R. A. M. C., Lt.-Colonel Joseph Blake, M. R. C., Médecin-Major Leriche, and Médecin-Major Heitz-Boyer.*

*The subject of the second meeting, Saturday July 27, at 10 : 00 A. M., was " The Sources of Wastage of Men in War : with Suggestions for their Amelioration ". It was discussed by Lieutenant-General Burtchaell, R. A. M. C., Colonel H. Ensor, R. A. M. C., Lt.-Colonel C. G. Brown, R. A. M. C., Médecin-Major Hautant, Major Cowie, R. A. M. C., Major-General W. M. Ireland, M. C., and Major Emerson, M. R. C.*

*The Subject of the third meeting, Saturday July 27, at 2 : 00 P. M. was " Transportation of Wounded Men ". It was discussed by Colonel J. S. Gallie, R. A. M. C., Colonel H. Ensor, R. A. M. C., Colonel J. Poe, R. A. M. C., Lt.-Colonel R. S. C. Thomson, Médecin-Major R. Proust, and Colonel Percy Jones, U. S.*

*Following the meeting there was a demonstration by Colonel Gilchrist of a degassing plant.*

### FRACTURES OF THE FEMUR

**Treatment of Fractures of the Femur.** By Major SINCLAIR, R. A. M. C. The following is a detailed résumé of the paper :

In the trenches.

*Arrest of Hemorrhage.* The tourniquet should be applied just sufficiently to control the hemorrhage. It must be removed at the earliest possible opportunity at the field ambulance or C. C. S. and here the vessels should be ligatured, as great harm is done by prolonged or unnecessary application of the tourniquet.

*Protection of the Wound.* Do not attempt to clean the wound but simply apply the first field dressing to protect it from further infection.

*Immobilize the Limb.* The Thomas is the simplest and most efficient splint, in which every fracture of the femur, leg, and foot can be treated uniformly with good results at the front and at the base.

Apply the Thomas splint over the clothes, get the ring against the ischial tuberosity, with one hand round the patient's ankle extend the limb, and at the same time push the end of the splint up with

Skewer Extension suint Suspended on Stocker Bar.

your abdominal muscles, so as to get and keep the ring pressed home. Take the skewer in the other hand and pass it obliquely from the inner side between the patient's sole and the sole of the boot, in such a way that when it is resting on the side bar the foot is rotated outward. This conforms with the external rotation of the lower end of the upper fragment which assumes a position of repose in all fractures, and by fixing the foot prevents the conlinual rotation of the upper end of the lower fragment during transit. This movement at the upper end of the lower fragment tays open fresh channels for infection, not to mention the discomfort of the patient. Place a cork over the sharp end of the skewer. Pass two loops of bandage around the projecting ends. Make a fixation pull, passing the external bandage below and the internal bandage above their respective side bars, and tie off with a V-shaped depression on the end of the splint.

Support the thigh posteriorly by a bandage, passing from bar to bar. Fix the knee in the splint by padding all around, and encircling the side bars with the same bandage. Then continue this bandage zig-zagging from bar to bar, to support the leg posteriorly and fix the ankle in the same way as at the knee. Finally tie the skewer to the side bar.

Transfer the patient to a stretcher and suspend the limb to stretcher bar. This prevents the limb from being driven forward out of the splint, as it is when laid on a horizontal surface, and avoids an angulation backwards at the seat of fracture, thus protecting the soft tissues in the vicinity of the break from further damage.

### Field Ambulance or C. C. S.

On arrival at the field ambulance or C. C. S., X-ray if possible and give A. T. S. Remove clothing, cutting it away from the injured limb and remove the boot. Although the temporary extension to the boot can be very quickly applied and is of great service for a few hours, it will cause pressure sores on the dorsum if the pull be excessive or kept on too long. Ankle ties only give extension and do not fix the foot. Having taken off the boot, tie a bandage around the ankle and make this fast to the V depression at the end of the splint.

### *Adhesive Glue.*

The following is a formula :

Very good glue, 50 parts;
Water, 50 parts;
Glycerine, 4 to 6 parts;
Menthol, 1 part.

The adhesive *per se* cannot cause blistering.

See that the temporary extensions are fixed and that the limb up to the field of operation is supported posteriorly.

*Operation.* The operation should be performed aseptically and, as Berkeley Mohynham says, "caress your tissues" with great gentleness. Do not make extensive excisions ; stop hemorrhage. Bone which is lying totally detached in the muscles may be removed, but bone with attachments try gently and carefully to return to its old bed. Keep in your track and you will find your foreign body and cloth, if present. Make a dependent counter-opening in the posterior aspect of the thigh, with limb raised and body lowered, so as to insure drainage by gravity. Insert a half inch rubber drainage tube, extending from the fracture in a slanting direction towards the buttock and projecting about one inch from the skin. Prove the drain with about two ounces of normal saline; henceforward

General View. First Position Extension by Screws in Tibia.

keep the wound dry and drain properly until the bone is repaired. Support the limb posteriorly with sterile flannel bands passed over the inner bar and fixed to the outer by sterile clips, the tube still being allowed to pass between.

*Primary Suture.* Should not be employed in evacuation cases. If sutured, cases must be carefully chosen and kept 14 days under the charge of the operating surgeon.

### Base.

It is necessary to have a mobile X-ray plant, splint-room workshop, clean and septic theaters, besides an electrical department. The staff should have a special aptitude for this class of work and should not be moved even within their unit, as the best results always come from the wards where the staff is permanent. The first thing of all, the patient is sent to the X-ray; when he returns to the ward the wounds are redressed with a small dry gauze dressing, and temporary extension is applied by means of a bandage tied round the ankle and made fast to the end of the splint, the limb is immobilized while it is being washed as at the C. C. S., and the extensions which were applied in a hurry are removed. The

Net Frame Showing How Dressing May Be Done.

Thomas splint is bent at the knee, extension prop fixed at the lower end, and four tape loops attached, two applied to the ring of the splint, below the side bars, and two to the side bars at the foot end.

*Method of Reduction.* If the case has a large buttocks wound the case is treated in a *Net Frame.* If the case has a marked swollen thigh, he is taken to the theatre and drained. All operations except amputations are done in the splint or net frame. To try and combat the shock after these operations, the author proposes to anastomose the femoral artery to the vein. The patient remains in the first position about seven weeks until the fractured ends are sufficiently held by callus and the limb is about one-half an inch longer than the other. The callus, if splastic, will bend gradually but will crack if bent suddenly, so angulations very frequently occur in the later stages. In large comminuted fractures with much bone loss the author has seen angulations occurring 18 months after injury. Comparative X-ray photographs ought to be taken monthly, which give very valuable information regarding bone degeneration, and destruction of sequestra.

## Figures and Tables

**Figure f1:**
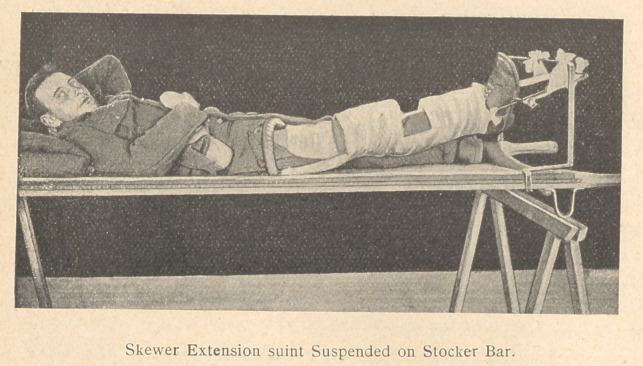


**Figure f2:**
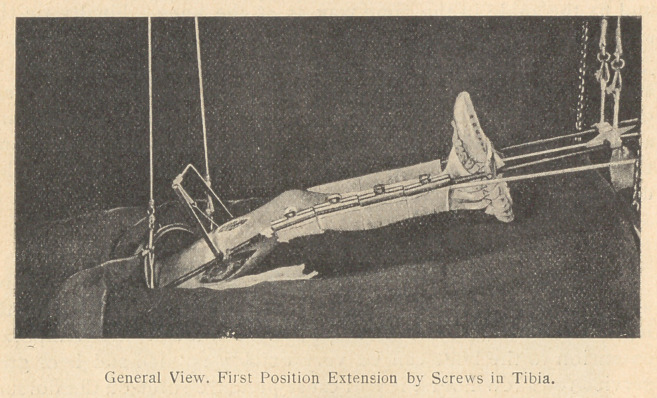


**Figure f3:**